# Long Non-Coding RNAs in Cancer and Development: Where Do We Go from Here?

**DOI:** 10.3390/ijms16011395

**Published:** 2015-01-08

**Authors:** Monika Haemmerle, Tony Gutschner

**Affiliations:** 1 Department of Gynecologic Oncology and Reproductive Medicine, the University of Texas MD Anderson Cancer Center, Houston, TX 77054, USA; E-Mail: mhaemmerle@mdanderson.org; 2 Institute of Pathology, University Hospital Heidelberg, Heidelberg 69120, Germany; 3 Department of Genomic Medicine, the University of Texas MD Anderson Cancer Center, Houston, TX 77054, USA

**Keywords:** functional genomics, genetically engineered mouse models (GEMM), long intergenic RNA (lincRNA), metastasis, metastasis-associated lung adenocarcinoma transcript 1 (MALAT1), HOX transcript antisense RNA (HOTAIR)

## Abstract

Recent genome-wide expression profiling studies have uncovered a huge amount of novel, long non-protein-coding RNA transcripts (lncRNA). In general, these transcripts possess a low, but tissue-specific expression, and their nucleotide sequences are often poorly conserved. However, several studies showed that lncRNAs can have important roles for normal tissue development and regulate cellular pluripotency as well as differentiation. Moreover, lncRNAs are implicated in the control of multiple molecular pathways leading to gene expression changes and thus, ultimately modulate cell proliferation, migration and apoptosis. Consequently, deregulation of lncRNA expression contributes to carcinogenesis and is associated with human diseases, e.g., neurodegenerative disorders like Alzheimer’s Disease. Here, we will focus on some major challenges of lncRNA research, especially loss-of-function studies. We will delineate strategies for lncRNA gene targeting *in vivo*, and we will briefly discuss important consideration and pitfalls when investigating lncRNA functions in knockout animal models. Finally, we will highlight future opportunities for lncRNAs research by applying the concept of cross-species comparison, which might contribute to novel disease biomarker discovery and might identify lncRNAs as potential therapeutic targets.

## 1. The Emergence of lncRNA

For more than five decades it has been known that DNA sequences are transcribed into RNA but never get translated into protein. This challenged the central dogma of molecular biology that put RNA as a simple messenger between the DNA and protein worlds. However, most researchers ignored these untranslated RNAs, assuming that they do not serve a useful purpose. In the mid-1990s, researchers like John Mattick started to argue that these RNAs transmit regulatory information, which might be associated with the emergence of multicellular organisms [1]. Only recently has this idea received strong support by the observation that the proportion of non-coding genomic sequences correlates broadly with the developmental complexity [2]. While this finding is still controversial and might be explained by an inefficient selection against non-functional genomic elements as body size increases and population size decreases [3], progress in the field of genome-wide sequencing technology and transcriptome analysis led to the astonishing notion that up to 70%–90% of the human genome is transcribed into RNA [4,5,6]. However, only 1%–2% of the human genome contains the blueprint for protein-coding transcripts, which led to the birth of a new category of transcripts—long non-coding RNAs (lncRNAs). Many of these lncRNAs are expressed in a tissue-specific and timely restricted manner and show a low level of expression and sequence conservation [7,8,9]. While “transcription” per se does not automatically equal “function”, research over the last decade has shown that lncRNAs can have important functions in developmental processes, influence differentiation, and play a role in human diseases, e.g., cancer [10] or neurodegenerative disorders like Alzheimer’s Disease [11,12]. Particularly interesting is the notion that about 95% of all variants associated with complex human diseases map to non-coding, presumably regulatory, sequences [13,14]. However, lncRNA research is still in its infancy and scientists are only beginning to unravel the molecular functions of these new transcripts. As in any other developing area, novel tools and model systems need to be developed first to allow in-depth understanding of molecular details. Bona fide animal models, *i.e.*, transgenic overexpression or knockout animals are the gold standard for functional genomics and are routinely used to analyze the function of protein-coding genes. However, recent genetic knockout experiments in mice have uncovered pitfalls that have to be considered, if the gene of interest is a non-coding RNA.

In this review, we will discuss the challenges associated with probing the *in vivo* function of lncRNAs in animal models. Moreover, we will highlight the power of cross-species comparisons and how this approach might be used to identify conserved lncRNAs with a role in human diseases, e.g., cancer.

## 2. LncRNA Knockout—One Aim, Multiple Options

**Figure 1:**
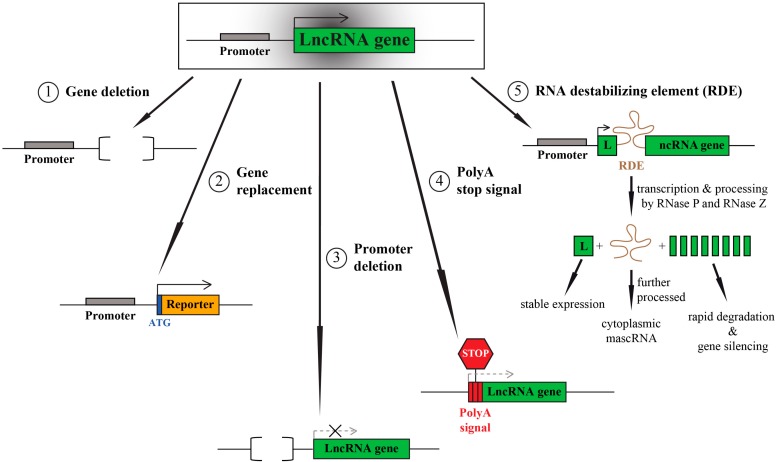
Long non-protein-coding RNA (LncRNA) targeting strategies for *in vivo* loss-of-function studies.

Several targeting strategies can be used for knocking out protein-coding genes: exon replacements, in frame stop-codon insertion or introduction of insertions that lead to a frame shift, as well as whole gene excisions, truncations or (point) mutations of functional domains. Most of these manipulations need active translation of the RNA transcript to achieve the disruptive effect, and thus are not feasible for lncRNAs. Hence, lncRNA targeting strategies must prevent the whole transcript from being made (Figure 1).

The easiest way to achieve this requires the deletion of the complete gene sequence which relies on homologous recombination, and can be used to generate constitutive or conditional knockout animals. We and our colleagues applied this strategy to generate constitutive *Malat1* knockout mice [15].

A related strategy replaces the lncRNA sequence with a reporter gene (monoallelic or biallelic), e.g., *LacZ*. If the endogenous lncRNA promoter is kept intact during the replacement, it can drive reporter gene expression thereby revealing lncRNA expression patterns *in vivo*. This strategy has been applied by John Rinn, Paolo Arlotta and co-workers in an impressive large-scale knockout project to investigate the phenotypes of 18 lncRNA knockouts in mice [16].

Instead of deleting or replacing the whole lncRNA it is also possible to just remove the endogenous promoter. The deletion can be small (only a few hundred base pairs) and might only minimally perturb the genomic locus (in contrast to the deletion of the complete gene). However, many protein-coding and non-coding genes have alternative promoters and hence will retain expression of one or more isoforms, if only one promoter is targeted. Moreover, divergent transcription of lncRNAs in close proximity to protein-coding genes is frequently observed [17,18,19]. In such a scenario promoter deletions may affect both genes and make interpretation of results more complicated. Promoter targeting strategies were used to target the neighboring lncRNAs *Neat1* and *Malat1* [20,21].

A third strategy for targeting lncRNAs is the integration of strong transcriptional stop signals at the very 5'-end of the non-coding transcript. The integration of polyadenylation (poly A) signals at the beginning of the transcript causes premature cleavage and polyadenylation of the lncRNA, and finally its degradation. This approach can yield strong, but also incomplete lncRNA depletion, depending on the lncRNA abundance. We successfully applied this strategy, in combination with integration of a selection marker (*Green Fluorescent Protein*, *GFP*) to silence the lncRNA *Malat1* in human cancer cells [22,23]. Other lncRNAs targeted by this approach include, e.g., *Airn* and *Evf2*, and this approach was chosen to generate a third *Malat1* knockout mouse model [24,25,26].

An additional and novel strategy could be the use of RNA destabilizing elements. In our previous study, we identified an efficient RNA destabilizing element derived from the 3'-end of the endogenous *Malat1* transcript [22]. This motif is recognized and processed by nuclear RNase P, which cuts at the 5'-end of the motif, and ultimately leads to the degradation of the downstream 3'-end of the RNA. In contrast, the upstream 5'-end of the transcript is stabilized due to the formation of a triple helical structure [27,28,29,30]. If inserted after the open reading frame of a reporter gene, this element also enables efficient protein translation (own data and [30]). The recognition of this motif by RNase P depends on its orientation and therefore should allow strand-specific silencing [22]. This is important, if the lncRNA is antisense to a protein-coding gene. Furthermore, Cre-mediated inversion of its orientation would enable a timely controlled gene silencing. In analogy to the polyA stop signal, integration of this *Malat1*-derived element at the beginning of a transcript would cause a cleavage and silencing of the downstream sequences. Importantly, this does not depend on RNA polymerase II and its associated factors, and thus represents an interesting strategy to target not only RNA polymerase II, but also I and III transcripts. However, to our knowledge this idea has not been tested so far *in vivo*. One thing that needs to be considered is the generation of a small ncRNA (mascRNA) during the cleavage and processing events. The tRNA-like mascRNA will be exported to the cytoplasm where it might have a so far unknown function [29].

## 3. LncRNA Knockouts—Things to Consider

The aforementioned strategies represent solutions to the problem of “How to target lcnRNAs?”, but they immediately raise another question: Which strategy is the “right” one? Unfortunately, there is no “one fits all” answer to this question. To make a good decision requires some prior knowledge about the lncRNA, its genomic and cellular localization, and its putative function. For example, many lncRNAs act as primary host gene for classes of small non-coding RNAs, e.g., microRNAs. Thus, deletion of the complete lncRNA locus will disrupt the function of multiple transcripts in parallel. Moreover, lncRNAs might regulate the expression of neighboring genes in *cis*, or of distant genes in *trans*. The lncRNA might achieve this via physical interaction with proteins or other nucleic acids. Hence, the RNA product is important and blocking its production via insertion of stop signals or other destabilizing elements (at the beginning of the transcript) might be the strategy of choice. Alternatively, the simple act of transcription through an lncRNA gene locus could be critical because of the induction of chromatin changes and/or modifications, or the recruitment of other proteins, e.g., transcription factors. In this case, the RNA transcript is only a by-product and modulation of the transcription event, e.g., by targeting the promoter, might be an appropriate strategy.

Consequently, multiple strategies might be considered and even used in parallel to help interpretation of phenotypic results. Minimal genomic disruptions should be used and control manipulations should be performed to distinguish between effects caused by the intended disruption of the lncRNA or the unintended disruption of neighboring genes or regulatory DNA elements with which lncRNA loci are interleaved across the entire genome. In the case of trans-acting lncRNAs, phenotypes should be rescued upon expression of the lncRNA from an independent transgene. The transgene should contain the endogenous regulatory sequences to maintain physiologically relevant lncRNA expression levels. Moreover, correct developmental timing as well as tissue- and cell-specific lncRNA expression patterns should be considered in these rescue experiments. Given the large size of some lncRNAs (up to several hundred kb) these experiments might be very challenging. In cases where lncRNAs act in *cis*, a combination of several targeting strategies might be required to unravel true lncRNA-dependent effects. For an in-depth description and detailed discussion about the advantages and challenges associated with current lncRNA knockout strategies the reader is referred to Bassett *et al.* [31].

## 4. LncRNAs in Development—Lack of Phenotypes besides High Conservation

Only a few lncRNA knockout studies have been conducted till today. Our own knockout study, together with the work of two other groups, targeting the lncRNA *Malat1* led to the finding that the loss of this lncRNA is compatible with life and development [15,21,25]. This finding was highly unexpected given the strong nucleotide sequence conservation in mammals, and the ubiquitous and abundant expression of *Malat1*. Moreover, *MALAT1* has been linked to several human cancers and was shown to regulate cell cycle progression, apoptosis, migration and metastasis of cancer cells [32,33]. On the molecular level *MALAT1* was shown to regulate alternative splicing of specific transcripts as well as the expression level of different genes, presumably via its interaction with chromatin modulators [33,34,35,36,37]. The lack of an overt phenotype under physiological conditions of the three published *Malat1* knockout mouse models that had been generated by different targeting strategies might be explained by functional redundancy or compensatory mechanisms. In the future, application of certain stresses or other pathological scenarios might help to reveal a phenotype. In this line, a recent study could show that *MALAT1* expression in endothelial cells is up-regulated under hypoxia and controls the phenotypic switch from migration to proliferation in endothelial cells *in vitro* and *in vivo* [38].

The example of *Malat1* is not the only case where the knockout model did not yield a discernable phenotype. Other knockout studies targeting broadly expressed lncRNAs (e.g., *Neat1*) or highly conserved regions of the mammalian genome did not result in developmental aberrations [20,39]. Remarkably, deletion of 13 out of 18 carefully selected lncRNA genes that were targeted by Sauvageau, Goff, Lodata *et al.* did not reveal a phenotype [16]. Altogether, these surprising findings might be explained by functional redundancies and compensatory mechanisms that might develop in constitutive knockout models, and the acute depletion of the lncRNA gene in conditional model systems might yield different outcomes. Moreover, many lncRNAs are primate-specific and a large fraction is expressed in the brain [8,40]. This suggests that much of the lncRNA-mediated genetic information is devoted to brain function. Therefore, besides histopathological examination of the animals’ organs and tissues, phenotypic analyses need to consider tissue-/cell-type specific lncRNA expression pattern, and need to include cognitive screens and a careful observation of the animals’ behavior.

## 5. LncRNAs in Cancer—Cross-Species Comparisons to Reveal Cancer Genes and Functions

Many lncRNAs are differentially expressed between normal and malignant cells, and initial *in vitro* experiments revealed a function of these lncRNAs in controlling cell cycle progression, apoptosis or migration. *Malat1* was one of the first lncRNA linked to human cancer [41]. Given *Malat1*’s well-established role in a broad range of human cancer cells and its newly identified role in the tumor microenvironment, *i.e.*, in endothelial cells, raises the exciting question: Will *Malat1* deletion impair tumor growth and progression in genetically engineered mouse models (GEMMs)? A straightforward way to answer this question is to cross the *Malat1*^−/−^ mouse with several of the relevant tumor models that show spontaneous metastasis, e.g., the RIP-Tag model of pancreatic islet carcinoma [42], the MMTV-PyMT, MMTV-Erbb2 and other models of breast cancer [43], or one of the *KRAS*-driven lung cancer models [44]. For a more detailed analysis, conditional *Malat1* knockout models would be needed to delete this lncRNA at a certain stage during the tumor development, or in a specific compartment of the tumor environment (endothelial cells *vs.* epithelial tumor cells). Alternatively, non-germline genetically engineered mouse models could be used as well offering flexibility, speed and uniformity at reduced costs [45].

These experimental strategies might be exploited for other de-regulated lncRNAs as well and are not limited to *Malat1*. For example, the expression of the lncRNA *Hotair* (HOX transcript antisense RNA) is increased in primary breast tumors and metastases, and its expression level in primary tumors is a predictor of eventual metastasis and survival [46]. *Hotair*^−/−^ mice are viable [47], and thus would enable functional and therapeutic studies in murine tumor models.

An alternative yet complementary approach to investigate the role of lncRNAs in cancer could take advantage of existing murine tumor models to analyze structural and sequence alterations (gene fusions, copy-number alterations, mutations), and to profile the expression of conserved lncRNAs during cancer progression. The obtained data could be integrated with data from human cancer patients to pinpoint recurrent and conserved genetic alterations that involve putative oncogenic or tumorsuppressive lncRNAs. These analyses could be extended to investigate mechanisms of therapy resistance that might be mediated by lncRNAs. However, the biggest challenge is the identification of conserved lncRNAs. In general, gene conservation is judged based on sequence similarities—either on the level of nucleotide or amino acid sequence. While this is useful for protein-coding genes it is not directly applicable for lncRNAs due to their less conserved nucleotide sequences [48,49]. For example, a comparison between lncRNAs expressed in mammals and zebrafish identified only a few significantly conserved sequences, mostly restricted to short sequence stretches [50]. Hence, lncRNAs evolve rapidly and often lack orthologs in other species. Therefore, additional dimensions of conservation need to be considered when working with non-coding RNAs [51].

Cross-species cancer gene analysis, which integrates multidimensional genome-wide cancer data sets from human and mice, represents a powerful approach for identifying and validating cancer-relevant genes [52,53]. Application of this concept to the field of lncRNAs has the potential to identify novel therapeutic targets and putative biomarkers.

## 6. Conclusions and Outlook

LncRNAs are more and more recognized as important regulators of diverse cellular processes and are actively involved in signaling pathways [54]. Leveraging the power of genome-wide sequencing techniques will generate a comprehensive catalogue of lncRNAs involved in human diseases, e.g., cancer. The development of novel assays to map lncRNA interactions with proteins and other nucleic acids will help to further investigate the molecular function of lncRNAs [34,55,56,57]. Novel assays might also be developed to study the interaction between lncRNAs and additional biomolecules, e.g., lipids or small second messengers (e.g., phosphatidylinositol-(3,4,5)-trisphosphate). Further insights into lncRNA conservation might come from newly developed genome-scale structure mapping techniques [58,59,60,61]. Integration of structural information and lncRNA interaction sites will guide future lncRNA targeting strategies. The use of novel genome editing tools, *i.e.*, the CRISPR/Cas9 system [62] will largely contribute to generate better *in vivo* and *in vitro* models for basic lncRNA research. Translational research efforts might benefit from these newly developed mouse models by integrating lncRNAs into disease modeling, and will generate valuable tools for preclinical testing of anti-lncRNA therapeutics. Effective therapeutics might target the lncRNA directly via small interfering RNA (siRNA) or antisense oligonucleotide (ASO) mediated silencing. Alternatively, lncRNA function might be inhibited indirectly via blocking the physical association with its interaction partners. Recent progress in the field of RNAi therapeutics will help to deliver these anti-lncRNA therapeutics *in vivo* [63,64].
